# The gaps in health-adjusted life Expectancy (HALE) by income and region in Korea

**DOI:** 10.1093/eurpub/ckac131.531

**Published:** 2022-10-25

**Authors:** YS Jung, YE Kim, M Ock, SJ Yoon

**Affiliations:** 1 Institute for Future Public Health, Korea University, Seoul, South Korea; 2 Department of Big Data Strategy, National Health Insurance Service, Wonju, South Korea; 3 Department of Preventive Medicine, University of Ulsan College of Medicine, Ulsan, South Korea; 4 Department of Preventive Medicine, Korea University College of Medicine, Seoul, South Korea

## Abstract

This study aims to calculate the health-adjusted life expectancy (HALE) by using years lived with disability (YLD) from the national claims data, as well as to identify the differences and inequalities in sex, income level and region. The study was carried out on total population receiving national health insurance and medical benefits. We calculated incidence-based YLD for 260 disease groups, and used it as the number of healthy years lost to calculate HALE. We adopted the insurance premium to calculate the income as a proxy indicator. For the region classification, we chose 250 Korean municipal-level administrative districts. The primary outcome was HALE in the Korean population. The second outcome was the HALE’s gap in terms of sex, income, and region. Our results revealed that HALE increased from 2008 (68.89 years) to 2019 (70.58 years). HALE in males increased faster than that in females. HALE was higher in higher income levels. In 2019, the gap in HALE between Q1 and Q2, the lower income group, was about 5.70 years. The gap in females by income level was smaller than that in males. Moreover, the gap in HALE by region was found to increase. Results suggest that there is an inequality in YLD in terms of income level in Korea. Therefore, we need intensive management for the low-income group to increase HALE at the national level.

**Key messages:**

• Males’ health level may be more sensitive to the socioeconomic level than females’ health level.

• In the 5th National Health Plan (HP2030), it was decided to set a target value for the overall goals based on this result.

